# Transition and continuity of care after hospital discharge for COVID-19 survivors

**DOI:** 10.1590/1980-220X-REEUSP-2023-0083en

**Published:** 2023-11-20

**Authors:** Aline Marques Acosta, Carlise Rigon Dalla Nora, Raquel Malta Fontenele, Gisele Knop Aued, Cristhiane de Souza Silveira, Amanda Xavier Sanseverino

**Affiliations:** 1 Universidade Federal do Rio Grande do Sul, Escola de Enfermagem, Departamento de Assistência e Orientação Profissional, Porto Alegre, RS, Brazil.; 2 Universidade Federal do Rio Grande do Sul, Escola de Enfermagem, Porto Alegre, RS, Brazil.

**Keywords:** Patient Discharge, Continuity of Patient Care, Coronavirus Infections, Quality of Health Care, Alta del Paciente, Continuidad de la Atención al Paciente, Infecciones por Coronavirus, Calidad de la Atención de Salud, Alta do Paciente, Continuidade da Assistência ao Paciente, Infecções por Coronavírus, Qualidade da Assistência à Saúde

## Abstract

**Objective:**

To assess care transition quality and compare it with the clinical characteristics and continuity of care after hospital discharge of COVID-19 survivors.

**Method:**

This is a descriptive, observational and cross-sectional study, carried out with 300 patients with COVID-19 who were discharged from a hospital in southern Brazil. The Care Transitions Measure (CTM-15) and question guide about symptoms, difficulties and use of health services after discharge were used. Student’s t-test, Pearson and Spearman correlation were used.

**Results:**

The mean score for care transition quality was 74.2 (±18.2). Factors associated with higher quality were receiving care in intensive care (p = 0.001), using non-invasive mechanical ventilation (p = 0.05), using vasopressors (p = 0.027) and having an appointment at the hospital after discharge (p = 0.014). Positive correlated factors were length of stay (p = 0.017), and negative factors were post-discharge symptoms of fatigue (p = 0.001), weakness (p = 0.008), difficulty doing moderate activities (p = 0.003) and how difficult recovery is (p = 0.003).

**Conclusion:**

Most participants had a satisfactory perception of care transition. However, aspects such as care plans, referrals and follow-up after hospital discharge require improvements.

## RESUMO

**Objetivo:**

Avaliar a qualidade da transição do cuidado e compará-la com as características clínicas e de continuidade do cuidado pós-alta hospitalar de sobreviventes de COVID-19.

**Método:**

Estudo descritivo, observacional e transversal, realizado com 300 pacientes com COVID-19 que tiveram alta de hospital do sul do Brasil. Utilizaram-se o *Care Transitions Measure* (CTM-15) e um roteiro de perguntas sobre sintomas, dificuldades e uso de serviços de saúde pós-alta. Utilizaram-se os testes t de Student, correlação de Pearson e Spearman.

**Resultados:**

O escore médio para a qualidade da transição do cuidado foi de 74,2 (±18,2). Fatores associados à maior qualidade foram ter atendimento em terapia intensiva (p = 0,001), usar ventilação mecânica não invasiva (p = 0,05), usar vasopressores (p = 0,027) e consultar no hospital após alta (p = 0,014). Fatores correlacionados positivos foram tempo de permanência (p = 0,017), e negativos, sintomas pós-alta de fadiga (p = 0,001), fraqueza (p = 0,008), dificuldade para fazer atividades moderadas (p = 0,003) e quão difícil é a recuperação (p = 0,003).

**Conclusão:**

A maioria dos participantes teve percepção satisfatória da transição do cuidado. Entretanto, aspectos como plano de cuidado, encaminhamentos e acompanhamento pós-alta hospitalar necessitam de melhorias.

## RESUMEN

**Objetivo:**

Evaluar la calidad de la transición asistencial y compararla con las características clínicas y de continuidad de la atención post alta hospitalaria para sobrevivientes de COVID-19.

**Método:**

Estudio descriptivo, observacional y transversal, realizado con 300 pacientes con COVID-19 que fueron dados de alta de un hospital del sur de Brasil. Se utilizó el Care Transitions Measure (CTM-15) y un guión de preguntas sobre síntomas, dificultades y uso de los servicios de salud post alta. Se utilizaron pruebas t de Student, correlación de Pearson y Spearman.

**Resultados:**

El puntaje promedio para la transición de la calidad de la atención fue 74,2 (±18,2). Los factores asociados con mayor calidad fueron recibir atención en cuidados intensivos (p = 0,001), utilizar ventilación mecánica no invasiva (p = 0,05), utilizar vasopresores (p = 0,027) y visitar el hospital después del alta (p = 0,014). Los factores correlacionados positivamente fueron el tiempo de estancia hospitalaria (p = 0,017), y los negativos, síntomas de fatiga después del alta (p = 0,001), debilidad (p = 0,008), dificultad para realizar actividades moderadas (p = 0,003) y la dificultad de la recuperación (p = 0,003).

**Conclusión:**

La mayoría de los participantes tuvo una percepción satisfactoria sobre la transición del cuidado. Sin embargo, aspectos como el plan de cuidados, las derivaciones y el seguimiento post alta hospitalaria requieren mejoras.

## INTRODUCTION

The COVID-19 pandemic, caused by the SARS-CoV-2 virus, impacted humanity in large proportions, as it advanced quickly and lethally and highlighted the fragility of health systems in several countries, impacting epidemiological, social, economic, political and cultural aspects^(1)^. Brazil is one of the countries with the highest number of infected people in the world; as of January 2023, there were 36,768,677 confirmed cases and 696,603 deaths^(2)^.

Although the majority of patients infected with SARS-CoV-2 are asymptomatic or present mild symptoms, such as fever, rhinorrhea and cough, and recover without the need for hospital admission, some may progress to serious clinical complications, with involvement of the pulmonary, neurological, cardiovascular, urinary, among others^(3)^. In Brazil, a study identified a COVID-19 hospital admission rate of around 6%, with significant variation in the different phases of the pandemic. Among admitted to hospital patients, 20% require care in the Intensive Care Unit (ICU)^(4)^.

While efforts are expended in hospitals to save lives, little attention has been paid to the care needs of survivors returning home^(5)^. These patients are potential candidates for developing post-intensive care syndrome (PICS) and decreased health-­related quality of life^(6)^. Many need to deal with their comorbidities^(4)^ and may present, during recovery at home, complications related to the disease itself, the decompensation of previous morbidities and the treatment instituted^(7)^. Furthermore, several consequences persist after discharge, such as fatigue, weakness, dyspnea, neuropathy/myopathy, anxiety and depression^(3,8)^.

Discharge from hospital to home is a period of risk for patients, who must deal with new health problems and changes in the care plan, with adverse events, medication errors, difficulties in scheduling appointments and post-discharge examinations, readmissions and use of emergency services^(9)^. In the context of a pandemic, with social isolation, overcrowded health services and service restrictions, patients may present different post-discharge needs^(10)^.

Therefore, care transition actions are important to ensure continuity of care for COVID-19 survivors, in order to contribute to the physical, cognitive and psychological recovery and quality of life of affected patients, avoiding readmission in periods when which hospitals are overcrowded^(7)^. Nurses are central professionals in conducting care transition and managing hospital discharge, being able to enable continuity of care and contribute to comprehensive care^(5,11)^.

However, carrying out care transition actions is a complex process, even in the best of circumstances in hospital institutions^(5)^. In the context of a pandemic, the challenges are exacerbated, as services are forced to review dehospital admission processes to reduce hospital stay time, increase bed turnover and reduce hospital overcrowding.

Although care transition is an internationally explored topic, the literature is still emerging^(12)^, with a lack of studies that specifically deal with patients with COVID-19 in Brazil. Therefore, this study aims to assess care transition quality and compare it with clinical characteristics and continuity of care after hospital discharge of COVID-19 survivors.

## METHOD

### Study Design

This is a cross-sectional study, in which STrengthening the Reporting of OBservational studies in Epidemiology (STROBE) guidelines were followed.

### Place

The study was carried out from February to November 2021 at a large general university hospital and a reference in highly complex care for patients with COVID-19 in southern Brazil.

### Population and Selection Criteria

Patients aged 18 or over, who remained admitted to hospital in an inpatient unit for a minimum period of 48 hours, with a confirmed diagnosis of COVID-19 and discharged from the hospital to their home were included. Patients who did not live in the city of Porto Alegre and the metropolitan region and those who remained admitted to hospital only in the emergency department were excluded. During data collection, if patients had cognitive or communication deficits that prevented them from responding to the survey, caregivers who accompanied the discharge process and recovery at home could be interviewed as a substitute respondent (proxy informant), as carried out in other studies^(13,14)^.

### Sample Definition

The sample calculation was performed using WinPEPI (Programs for Epidemiologists for Windows) version 11.43. Considering an estimated population of 1,250 patients, obtained by weekly mean of hospital admissions for COVID-19 in the hospital studied, a 95% confidence level and a 5% margin of error, a minimum total of 295 participants was obtained. During the data collection period, 729 patients who met the inclusion and exclusion criteria were identified based on weekly reports from the computerized hospital management system. Of these, 353 (48.4%) did not respond to telephone contact in three attempts on different days and shifts of the same week; 20 (2.7%) did not agree to participate in the study; 54 (7.4%) were readmitted at the time of telephone contact; one (0.1%) was institutionalized; and one (0.1%) died after discharge. 250 (83.3%) patients and 50 (16.7%) caregivers responded to the survey.

### Data Collection

Data collection was carried out in two stages. The first stage took place from February to October 2021 through telephone contacts, 7 to 14 days after patients were discharged from the hospital. The Care Transitions Measure (CTM-15) was used, developed in the United States to assess care transition quality from patients’ and caregivers’ perspective^(15)^, which was adapted and validated for use in Brazil^(14)^. It consists of 15 items, which are organized into four factors, namely: Health management preparation; Medication understanding; Important preferences; and Care Plan^(14)^. Answer options are arranged on a Likert-type scale, in which a score is assigned according to participants’ response, as follows: totally disagree = 1 point; disagree = 2 points; agree = 3 points; totally agree = 4 points. There is also an option, “do not know/do not remember/not applicable”, which does not receive a score, as it is not included in the calculation of the final score. To calculate the mean score, according to the authors of the instrument, a formula is applied that transforms the results into scores from 0 to 100, with the higher the score obtained, the better care transition^(15)^. CTM-15 has been extensively tested and has proven to be reliable, accurate and valid for its purpose^(9,12,14)^.

Also, questions were asked about symptoms, difficulties and use of health services after hospital discharge, following a structured script drawn up based on the literature^(1,5,7)^ and the authors’ experience. It is noteworthy that, as this is an emerging topic, there is no validated questionnaire to identify symptoms and continuity of care for COVID-19 patients post-discharge. Therefore, a pilot study was carried out with 10 patients, who were not included in the sample. The script is structured and organized as follows: 13 questions about COVID-19 symptoms and difficulties after hospital discharge on a frequency scale that varies from “all the time” to “none of the time”; a question about the perception of the difficulty of recovery at home on a scale with answer options ranging from “not at all” to “extremely”; and seven questions about use of health services after discharge, with answer options of “yes”, “no” and “do not know/do not remember”. The items that deal with symptoms were scored on a scale of 0 to 4, as follows: no part of the time = 0 points; a small part of the time = 1 point; some of the time = 2 points; most of the time = 3 points; and all the time = 4 points. The item asking about how difficult recovery was scored as follows: not at all = 0; a little = 1; moderately = 2; enough = 3; extremely = 4. The remaining items about use of health services were coded: yes = 1; no = 2; and do not know/do not remember = 99.

The second stage of data collection was carried out in November 2021. Patients’ electronic medical records were consulted to identify sample characterization data, such as sex, age, marital status, race/color, education, comorbidities (according to ICD-10), length of stay in hospital, ICU admission, use of mechanical ventilation, vasopressors and/or dialysis method as well as the presence of emergency service or readmission within 30 days after hospital discharge. It is justified to collect this data after the telephone contact to have a difference of 30 days and obtain the readmission data of the last participant interviewed.

### Data Analysis and Treatment

Data were exported to an Excel spreadsheet and analyzed using the Statistical Package for the Social Sciences (SPSS) version 21.0. Regarding data analysis referring to CTM-15, the simple mean response for each item was calculated as well as the mean of the total scale and by factor, using the formula indicated by the authors that transforms the means into scores from 0 to 100^(15)^.

Regarding sample characterization, symptoms and continuity of care after hospital discharge, quantitative variables were described by mean and standard deviation or median and interquartile range. Categorical variables were described by absolute and relative frequencies.

Variable normality was assessed using the Kolmogorov-Smirnov test. Student’s t-test was used to compare means, and Pearson or Spearman correlation was used for the association between numerical and ordinal variables. The significance level adopted was 5% (p < 0.05).

### Ethical Aspects

The research was approved by the institution’s Research Ethics Committee, under Opinion 4,462,671/2020, in accordance with Resolution 466/2012. An Informed Consent Form (ICF) was used, with verbal consent from participants at the time of telephone contact for data collection and sending an electronic copy of the ICF by text message.

## RESULTS

In this study, it was observed that 168 (56%) patients were men, 135 (45%) were married, 247 (82.3%) were white, 99 (33%) had completed high school and had a mean age of 51.93 years. (±14.02). The most frequent comorbidities were hypertension (133; 44.3%), obesity (104; 34.7%), Diabetes Mellitus (78; 26%), asthma (28; 9.3%) and cancer (24; 8%). It was identified that 144 patients (48%) required ICU care. A total of 99 patients (33%) underwent invasive mechanical ventilation, and 205 (68.3%) non-­invasive mechanical ventilation. A total of 83 (27.7%) patients used vasoactive medications and 15 (5%) used dialysis. The median length of stay in hospital was 13 days (8-26). Only 10 (3.3%) patients were readmitted within 30 days after discharge.

Regarding care transition quality at discharge, the mean CTM-15 score was 74.2 (±18.2). Factor 1 (Health management preparation) obtained a mean score of 77.3 (±19.0); factor 2 (Medication understanding) obtained a mean score of 76.8 (±21.0); factor 3 (Important preferences) obtained a mean score of 76.1 (±18.3); and factor 4 (Care plan) obtained a mean score of 58.4 (±30.9). The item with the highest score was 14 (Understands how to take medications) and the lowest was 12 (Had written list of appointments and tests) (Table 1).

**Table 1:** Mean and standard deviation of Care Transitions Measure (CTM-15) item scores – Porto Alegre, RS, Brazil, 2021.

Item	Factor	Mean ± SD
1. Agreed health goals and means	3	80.8 ± 19.1
2. Preferences deciding health care needs	3	76.4 ± 21.3
3. Preferences deciding where needs met	3	72.2 ± 27.4
4. Had information needed for self-care	1	80.7 ± 21.6
5. Understands how to manage health	1	81.0 ± 20.6
6. Understand signs and symptoms	1	78.8 ± 22.2
7. Had written care plan	4	61.9 ± 33.3
8. Understand what makes better or worse	1	75.9 ± 25.2
9. Good understanding of things I was responsible for	1	79.0 ± 22.1
10. Confident I knew what to do	1	74.6 ± 24.2
11. Confident could do what needed	1	73.4 ± 25.2
12. Had written list of appointments and tests	4	54.7 ± 36.6
13. Understand medications’ purpose	2	80.6 ± 20.3
14. Understand how to take medications	2	84.2 ± 18.1
15. Understand medications’ side effects	2	66.7 ± 33.1

SD = standard deviation.

Regarding continuity of care, it was identified that 192 (64%) patients had some contact (via phone, message, email, home visit) with a health professional after discharge. However, 16 (5.3%) received a visit from a community health workers at home; 46 (15.3%) received care at the primary care reference unit; 5 (1.7%) needed emergency care; 36 (12.1%) had an appointment at the hospital’s outpatient clinic; and 30 (10%) had an appointment at a clinic or private office.

Furthermore, regarding recovery at home after hospital discharge, it was found that it was a little difficult for 85 (28.4%) patients, moderately difficult for 91 (30.4%), quite difficult for 24 (8%) and extremely difficult for 8 (2.7%). Table 2 shows the occurrence of symptoms after discharge, the most common being fear of reinfection and difficulty climbing several flights of stairs and difficulties in carrying out moderate activities such as moving a table, using a vacuum cleaner and sweeping the house.

**Table 2:** Frequency of symptoms 14 days after hospital discharge – Porto Alegre, RS, Brazil, 2021.

Post-discharge symptoms	All the time n (%)	Most of the time n (%)	Some of the time n (%)	Small part of the time n (%)	No part of the time n (%)	Do not know/not applicable n (%)
Fatigue	12 (4.0)	41 (13.7)	51 (17.0)	94 (31.3)	102 (34.0)	0 (0.0)
Weakness	18 (6.0)	27 (9.0)	59 (19.7)	94 (31.3)	102 (34.0)	0 (0.0)
Fever	0 (0.0)	2 (0.7)	2 (0.7)	21 (7.0)	273 (91.0)	2 (0.7)
Sore throat	5 (1.7)	4 (1.3)	10 (3.4)	27 (9.0)	252 (84.3)	1(0.3)
Dyspnea	4 (1.3)	6 (2.0)	35 (11.7)	65 (21.7)	189 (63.0)	1 (0.3)
Cough	5 (1.7)	18 (6.0)	48 (16.0)	97 (32.3)	131 (43.7)	1 (0.3)
Tachycardia	2 (0.7)	8 (2.7)	30 (10.0)	60 (20.0)	194 (64.7)	6 (2.0)
Agnosia	11 (3.7)	14 (4.7)	19 (6.3)	35 (11.7)	219 (73.0)	2 (0.7)
Diarrhea and/or vomiting	3 (1.0)	4 (1.3)	7 (2.3)	37 (12.3)	247 (82.3)	2 (0.7)
Dysphagia	4 (1.3)	2 (0.7)	7 (2.3)	18 (6.0)	266 (88.7)	3 (1.0)
Difficulty doing moderate activities	41 (13.7)	32 (10.7)	47 (15.7)	49 (16.4)	60 (20.1)	70 (23.4)
Difficulty climbing several flights of stairs	51 (17.0)	27 (9.0)	29 (9.7)	49 (16.3)	45 (15.0)	99 (33.0)
Fear of reinfection	75 (25.0)	43 (14.3)	53 (17.7)	54 (18.0)	68 (22.7)	7 (2.3)

Regarding the bivariate analysis of clinical variables in relation to the mean CTM-15 score, it was identified that caregivers had a worse perception of quality in care transition, with a mean CTM-15 score lower than that of patients (Table 3). Furthermore, receiving care in the ICU, using non-invasive mechanical ventilation and using vasopressors were associated with a higher mean of CTM-15. It was observed that the longer the length of stay, the higher the CTM-15 score.

**Table 3:** Bivariate analysis of clinical variables in relation to the mean Care Transitions Measure (CTM-15) score – Porto Alegre, RS, Brazil, 2021.

Variables	CTM-15	
	Mean ± SD	p
**Interviewee**		<0.001*
Patient	76.1 ± 17.5	
Caregiver	64.5 ± 18.6	
**Sex**		0.239*
Female	72.8 ± 18.5	
Male	75.3 ± 17.9	
**Age** (years)	r = – 0.095	0.101**
**Morbidities (CID-10)**		
**Hypertension**		0.571*
Yes	73.5 ± 19.2	
No	74.7 ± 17.4	
**Obesity**		0.494*
Yes	75.2 ± 17.5	
No	73.7 ± 18.6	
**Diabetes Mellitus**		0.683*
Yes	73.5 ± 20.0	
No	74.4 ± 17.5	
**Asthma**		0.374*
Yes	71.3 ± 20.9	
No	74.5 ± 17.9	
**Cancer**		0.747*
Yes	73.0 ± 17.8	
No	74.3 ± 18.2	
**Intensive Care Unit care**		0.001*
Yes	77.6 ± 17.2	
No	71.0 ± 18.5	
**Use of invasive mechanical ventilation**		0.084*
Yes	76.8 ± 17.4	
No	72.9 ± 18.5	
**Use of non-invasive mechanical ventilation**		0.05*
Yes	75.6 ± 17.8	
No	71.2 ± 18.7	
**Use of vasopressor**		0.027*
Yes	77.9 ± 17.5	
No	72.7 ± 18.3	
**Use of dialysis method**		0.096*
Yes	81.8 ± 18.3	
No	73.8 ± 18.1	
**Length of stay**	rs = 0.137	0.017***

CTM-15 = Care Transitions Measure; r = Pearson correlation coefficient; rs = Spearman correlation coefficient; *Student t-test; **Pearson correlation; ***Spearman correlation; SD = standard deviation.

In the bivariate analysis of variables relating to continuity of care, there was a weak negative correlation between the CTM-15 score and variables fatigue, weakness, difficulty in carrying out moderate activities and how difficult post-discharge recovery is (Table 4). Furthermore, having a hospital appointment after discharge was associated with a higher CTM score.

**Table 4:** Bivariate analysis of care continuity variables in relation to the Care Transitions Measure (CTM-15) score mean – Porto Alegre, RS, Brazil, 2021.

Variables	CTM-15	
	Mean ± SD	p
**Post-discharge symptoms**		
Fatigue	rs = – 0.192	0.001*
Weakness	rs = – 0.153	0.008*
Fever	rs = 0.008	0.888*
Sore throat	rs = – 0.014	0.813*
Dyspnea	rs = – 0.095	0.099*
Cough	rs = – 0.034	0.561*
Tachycardia	rs = – 0.068	0.244*
Agnosia	rs = – 0.048	0.406*
Diarrhea and/or vomiting	rs = – 0.065	0.260*
Dysphagia	rs = 0.014	0.816*
Difficulty doing moderate activities	rs = – 0.197	0.003*
Difficulty climbing several flights of stairs	rs = – 0.081	0.250*
Fear of reinfection	rs = 0.070	0.229*
**How difficult recovery is**	rs = – 0.172	0.003*
**Had contact (via phone, text, email, home visit) with a health professional**		0.293**
Yes	74.8 ± 18.3	
No	72.8 ± 17.9	
**Received a visit from a community health worker at home**		0.919**
Yes	74.6 ± 18.9	
No	74.2 ± 18.2	
**Had care at a reference health unit**		0.888**
Yes	73.6 ± 18.3	
No	74.3 ± 18.2	
**Had care in another health unit (other than a reference)**		0.643**
Yes	71.0 ± 21.6	
No	74.3 ± 18.1	
**Needed emergency care**		0.54**
Yes	74.3 ± 18.2	
No	74.2 ± 18.2	
**Had an appointment at the hospital outpatient clinic**		0.014**
Yes	81.3 ± 16.7	
No	73.4 ± 18.1	
**Had an appointment at a clinic or private office**		0.145**
Yes	68.2 ± 19.5	
No	75.0 ± 17.9	
**Readmission within 30 days**		0.580**
Yes	79.2 ± 16.0	
No	74.0 ± 18.3	

CTM-15 = Care Transitions Measure; r = Pearson correlation coefficient; rs = Spearman correlation coefficient; *Spearman correlation; **Student’s t-test; SD = standard deviation.

## DISCUSSION

This study is a pioneer in assessing care transition quality at hospital discharge for COVID-19 survivors and comparing it with clinical and continuity of care characteristics. The results reflect patients’ and caregivers’ opinion about the transition made in the hospital to return home and the difficulties in following up care in health services in the context of a pandemic.

Findings regarding sex, age and comorbidities are also consistent with other studies with COVID-19 survivors described in the literature^(3,16)^. ICU admission, use of mechanical ventilation and length of hospital stay rates were higher than the values found in São Paulo^(3)^ and the United States^(10)^. However, in addition to the differences in the social, economic and health resources of these locations, the studies mentioned collected their data in 2020, while this research was developed in 2021, period in which the Gamma variants circulated, with a higher hospital admission rate, and Delta, with a higher ICU care rate^(4)^.

The findings presented in this study showed that the main symptoms after hospital discharge were difficulty climbing several flights of stairs and difficulties performing moderate activities. Another study also highlighted some persistent ­post-COVID-19 complications, such as physical exhaustion, dyspnea and fatigue, joint pain, muscle pain or weakness, head­ache, sleep disturbances, dizziness, anxiety and depression^(1)^. Therefore, it is essential to be alert to the health situation of COVID-19 survivors so that they do not present situations that are harmful to the functioning of the biological system.

Furthermore, the percentage of patients who had hospital readmission is lower than that found in investigations with COVID-19 survivors^(3,10)^. However, it is important to highlight that 7.4% of the individuals contacted in this study were readmitted to the hospital during the data collection period and were excluded, which may perhaps underestimate the readmission rate identified.

It was also evident that the majority of participants had a positive perception of care transition at hospital discharge, according to CTM-15. Although the instrument does not have a predefined cut-off point, values above 70 are satisfactory^(13)^. Therefore, the results of this research indicate a satisfactory care transition quality for COVID-19 survivors, corroborating Brazilian studies with cancer patients^(12)^ that found similar CTM-15 scores. Higher values were found with pediatric patients^(17)^ and lower values with older adults^(18)^.

It can be identified that the factors that deal with health management preparation, medication understanding and important preferences obtained satisfactory scores and with few differences in the means. The literature points out that these aspects are fundamental for a safe and effective care transition^(5,9,11)^. An evidence-based model was recommended for use with ­COVID-19 patients upon discharge from hospital to go home, including several actions that ensure patient-centered care, with preferences and goals taken into account in care plan as well as health education of patients and caregivers for symptom management and treatment compliance^(5)^. Despite the limitations imposed by the pandemic, such as restricted visits, which prevent the provision of guidance on care for caregivers throughout hospital admission, health teams used communication and information technologies to develop discharge education actions, such as online appointments, educational videos and podcasts, video calls, among others^(19)^.

On the other hand, the factor that deals with the care plan and referrals after hospital discharge was assessed as unsatisfactory by participants. Items 12 (Had written list of appointments and tests) and 7 (Had written care plan) received the lowest mean. A similar result was found in a study with older adults^(18)^. Lack of discharge planning, absence of protocols or systematized counter-referral instruments, little coordination and communication between services are weaknesses reported in Brazil^(20,21)^. Furthermore, many hospitals do not have a care transition program or institutional documents that guide discharge plan preparation^(20,22)^. Therefore, discharge planning activities depend on the individual efforts of nurses, which does not happen in the context of a systematized plan^(21)^. Therefore, the need for strategies to overcome this gap and provide continuity of care after discharge is reinforced.

It is noteworthy that, in this study, caregivers had a lower CTM-15 score than patients, indicating a worse perception of quality in care transition. In another care context, such as caregivers of patients with stroke sequels, caregivers had difficulties with post-discharge demands, which were related to weaknesses in care transition^(23)^. Weak transitions are associated with greater burden on caregivers^(24)^. Therefore, it is essential to include family members as early as possible in discharge planning, in order to improve care transition quality.

The literature is clear in stating that people with compromised health status may have a worse care transition quality^(18)^, considering that patients admitted to the ICU and with a longer hospital stay have a worsening in their health status and quality of life three months after being discharged^(3)^. However, this study identified that a better CTM-15 score is associated with patients treated in the ICU, using non-invasive mechanical ventilation and vasopressors, correlated with length of stay and indicating that critical patients with longer hospital admission have a better perception of care transition quality. This can be justified by the greater time dedicated by health professionals to prepare the discharge of these patients with long hospital admissions, as they require greater care and attention.

On the other hand, it was found that patients with more symptoms of fatigue, weakness and difficulty performing moderate activities had lower quality scores. Furthermore, the more difficult the self-reported post-discharge recovery, the lower the CTM-15 score. These data suggest that patients with post-­discharge difficulties have a worse care transition quality. Therefore, outpatient follow-up after discharge is important to identify difficulties and monitor treatment and home care^(25)^.

It is noteworthy that in this study few patients received care at the primary care reference unit or consulted at the hospital outpatient clinic, clinic or private office after discharge, which demonstrates the need to improve elements of care transition, such as articulation and communication between the hospital and other services in the Health Care Network, in order to promote continuity of patient care. In a study in the United States, it was identified that only 26.8% and 1.6% of ­COVID-19 survivors had a scheduled appointment in primary care and with a specialist at the time of discharge, respectively^(10)^.

In this study, it was observed that those with a hospital appointment after discharge had a better perception of care transition quality. Another investigation found that higher care transition scores were associated with higher rates of ­follow-up appointment in primary care^(26)^. Therefore, despite numerous difficulties in carrying out post-discharge follow-up, it is recognized that follow-up through telephone contact, home visits and/or appointment in primary care can avoid hospital readmission and emergency care^(27,28)^.

However, this study has some limitations. First, it was carried out in a single hospital in the south of the country, therefore, it cannot represent the Brazilian reality. Second, 48.4% of eligible patients did not respond to telephone contact, a problem reported to be frequent in COVID-19 patients and is inherently associated with selection bias^(3)^. Third, it is important to consider that the results of care transition assessment using CTM-15 may have been influenced by participants’ feeling of gratitude for the health service^(17)^. Finally, questions about symptoms, difficulties and use of health services after hospital discharge are not part of a validated instrument.

In relation to advances in nursing, this study presents findings that point out gaps in the care transition process, such as discharge plan elaboration and primary and secondary care follow-up, which can be strategically worked on by researchers, managers and nurses, in order to advance care transition in Brazil.

## CONCLUSION

It was evident that care transition quality for COVID-19 survivors was satisfactory, according to CTM-15. However, aspects that require improvement were identified, mainly with regard to the care plan and referrals after hospital discharge. Most patients had contact with a health professional after discharge, but few received care in the primary care unit, in the hospital outpatient clinic or in a private office. Caregivers had a worse perception of care transition quality than patients. Clinical aspects associated with care transition quality were receiving care in the ICU, using non-invasive mechanical ventilation and using vasopressors. Regarding variables related to continuity of care, patients who had an outpatient appointment at the hospital after discharge had higher scores. On the other hand, it was found that the greater the frequency of symptoms of fatigue, weakness and difficulty performing moderate activities, the lower the quality score. Furthermore, the more difficult the self-reported post-discharge recovery, the lower the CTM-15 score.

It is recommended to develop strategies for implementing discharge planning, using a systematized protocol or instrument to prepare an individualized, written discharge plan. The healthcare team should involve patients and their caregivers in developing the plan, providing written instructions and guiding them through necessary referrals for home care. Furthermore, follow-up by telephone or visit is suggested within two weeks after discharge to clarify doubts and monitor difficulties in recovery at home, contributing to care transition.
